# Social distancing in public transport: mobilising new technologies for demand management under the Covid-19 crisis

**DOI:** 10.1007/s11116-021-10192-6

**Published:** 2021-04-22

**Authors:** Daniel Hörcher, Ramandeep Singh, Daniel J. Graham

**Affiliations:** Transport Strategy Centre, Department of Civil and Environmental Engineering, Imperial College London, London, UK

**Keywords:** Covid-19, Public transport, Demand management, Inflow control, Pricing, Auctioning, Tradeable permit schemes

## Abstract

Dense urban areas are especially hardly hit by the Covid-19 crisis due to the limited availability of public transport, one of the most efficient means of mass mobility. In light of the Covid-19 pandemic, public transport operators are experiencing steep declines in demand and fare revenues due to the perceived risk of infection within vehicles and other facilities. The purpose of this paper is to explore the possibilities of implementing social distancing in public transport in line with epidemiological advice. Social distancing requires effective demand management to keep vehicle occupancy rates under a predefined threshold, both spatially and temporally. We review the literature of five demand management methods enabled by new information and ticketing technologies: (i) inflow control with queueing, (ii) time and space dependent pricing, (iii) capacity reservation with advance booking, (iv) slot auctioning, and (v) tradeable travel permit schemes. Thus the paper collects the relevant literature into a single point of reference, and provides interpretation from the viewpoint of practical applicability during and after the pandemic.

## Introduction

The global pandemic Covid-19 imposes an unprecedented challenge on public transport operators. Most of these challenges are stemming from low demand levels and disturbances in the financial stability of operations. Demand for public transport services has dropped substantially because

(i) travel demand in general fell sharply due to lockdown measures and restrictions on a range of communal activities, including working at regular work places;
(ii) official restrictions on public transport use, specifically, might have been in place, due to the threat of violating social distancing rules on board or in stations; and
(iii) even if such official restrictions are lifted, travellers might be reluctant to use public transport services, due to the degree of perceived risk of infections in shared vehicles or during other stages of a journey in public areas.

An immediate consequence of the drop in demand is financial instability. Public transport operators rely on demand dependent cash inflows to a varying extent. Most of the densely used urban rail systems cover more than half of their operating costs by fare revenues, while other services rely almost exclusively on public subsidies (see Monchambert et al., forthcoming). On the other hand, the operating costs of public transport operators are mostly not demand dependent, which means that a quick adjustment of supply levels and production costs is impossible in response to the external shock. Even if capacity supply (e.g. the number of timetabled commercial runs) can be reduced compared to pre-pandemic levels, the capital cost of infrastructure and vehicle ownership remains fixed in the short run.

Under regular conditions, it is a popular view among managers of transport providers that the higher the self-financing ratio of an operator, the more stable its financial status will become in the long run. In the present situation we observe that self-financing has become a disadvantage; those service providers that are more reliant on public subsides and alternative forms of funding are less affected by the pandemic, at least in the short run. In the medium to long run, the latter financing model cannot provide guarantees either, as the pandemic is also likely to take its toll on local and central government budgets, which may in turn result in a financial famine, especially if subsidies are determined in the political process on the basis of present and prognosed ridership.

These immediate economic consequences are coupled with general uncertainty regarding the speed and shape of recovery after the pandemic. Little is known about ‘life after Covid-19’. Some commentators envision that ‘life will never come back to normal’, hinting that working from home will remain persistent after the crisis, and the traditional offices and work places in high-density urban areas will not be utilised the same way as before. In practice, this implies a permanent reduction in travel demand, and fewer commuting trips in particular to traditional employment centres where public transport was the backbone of everyday mobility. Note that this scenario would lead to a fundamental rearrangement of urban land use and the spatial structure of economic activity, beside the obvious transformations in the transport market.^1^ Others argue that severe health crises happened regularly over past centuries, including a number of infectious diseases with much higher human tolls than the present one. However, the urban culture has not disappeared, and cities always returned to the exploitation of the benefits of physical proximity that the urban economics literature attributes to sharing, matching and learning benefits (Duranton and Puga 2004, Graham et al., forthcoming). The future development path of public transport remains uncertain within these two extrema.

Public transport demand is more likely to be impacted by Covid-19 than other transport modes, including walking, cycling and private motorised mobility, the core reason being that physical proximity is harder to avoid in public transport. Under regular operating conditions, physical proximity is the main source of density and scale economies in public transport, which are the engines of its efficiency gains compared to other modes when large travel volumes enable vehicle sharing. Even though clear empirical evidence on the exact infection rate in pubic transport is currently unavailable, and this number is expected to be extremely context dependent, it is likely that the risk of infections increases with the occupancy rate of shared vehicles. That is, the industry faces a delicate trade-off: too high demand leads to the violation of social distancing rules and increased infection risk (combined with the degradation of the public image of the service), while too low demand endangers the financial sustainability of operations. This delicate trade-off generates a need for sophisticated demand management tools under Covid-19. Gkiotsalitis and Cats (2020b) review potential service planning strategies under the Covid-19 crisis, while the present paper contributes with an elaboration of demand management measures and their feasibility.

The working hypothesis of this paper is that public transport operators will have to maintain certain levels of social distancing in otherwise densely used stations and vehicles in the aftermath of the pandemic. In practice, this is equivalent to managing demand such that the occupancy rate of vehicles and other facilities never exceeds a predefined threshold. This is a novel task in public transport, because under regular operating conditions, urban buses and trains can be used up to their physical capacity, i.e. the operator does not need to prevent passengers to board a vehicle when capacities are not fully utilised.

The paper approaches this practical but highly policy relevant problem with in-depth reviews of available knowledge in the literature. The review is split into three parts. First, in Section 2 we explore recent epidemiological findings on the transmission paths of Covid-19 and the likelihood of infections in public transport. The related discussion is supported by early empirical evidence and policy interventions in response to the crisis. Second, Section 3 introduces the key challenges of social distancing in public transport networks from a theoretical point of view. Third, Section 4 elaborates on the prospects of five travel demand management methods in achieving social distancing. These are (i) inflow control with queueing, (ii) time and space dependent pricing, (iii) capacity reservation with advance booking, (iv) slot auctioning, and (v) tradeable travel permit schemes. The conclusions of our review are summarised in Section 5.

## Existing evidence and early policy response

Prior to the onset of the Covid-19 pandemic, epidemiological studies on other similar pathogens have reported that virus transmission in public transport is possible, and occurs as a result of physical proximity to infected persons in an enclosed space (Furuya 2007; Cui et al. 2011; Troko et al. 2011; Mohr et al. 2012; Browne et al. 2016; Gosce and Johansson 2018). Recent research on SARS-Cov-2, the virus pathogen that causes the Covid-19 disease, indicates that a number of potential transmission paths are possible, including: respiratory and direct contact with large droplets, airborne respiratory with small droplets (aerosols), fomite, fecal-oral, and ocular (van Doremalen et al. 2020; Chu et al. 2020).

A limited number of empirical epidemiological studies have been undertaken to establish whether there is an association between public transport use and infection with SARS-Cov-2. A series of studies employing regression or bivariate correlation analysis methods have been undertaken, and most studies report statistically significant associations between the use of public transport and case incidence. However, it should be noted that in this work, non-causal analysis methods are adopted, and the results may be influenced by confounding factors or omitted variables. For example, in early studies (Zhao et al. 2020; Zheng et al. 2020), socio-demographic and other spatial and physical attributes of origin and destination cities are not considered, but could have an effect on the reported results. There could also be potential discrepancies in correct detection of virus infection status.

- In early work, Zhao et al. (2020) undertake linear regression and find a statistically significant association between case incidence and train travel in Chinese cities, while associations with travel by car and air are found to be insignificant. Zheng et al. (2020) report significant positive linear correlations between trip frequency and daily and cumulative case incidence in China for air, bus, and train modes.
- Gaskin et al. (2021) develop negative binomial and Cox regression models for US counties, controlling for socio-demographic characteristics including ethnicity, population, population density, household size, and poverty status. It is found that the greater levels of public transport usage by adults is associated with an increase in case incidence and deaths. The authors acknowledge that though key socio-demographic attributes have been controlled for, there may be other confounding factors that have not been considered.
- Noland (2021) uses a fixed effects regression modelling framework to quantify the association between mobility and the virus reproduction number in the US. After accounting for fixed time-invariant and time-varying effects, it is found that a higher degree of mobility observed at transit stations is associated with a higher reproduction number, and hence greater virus transmission. Again, other confounding factors may be present, and the author advises that the results should be interpreted with care.

A small number of observational studies have been undertaken to calculate infection rates after exposure to an index case on ground based public transport modes. It should be noted that these studies again do not employ casual methods and do not account for other potential sources of exposure prior to or after the transit trip that could influence the results.

- Hu et al. (2021) quantify infection rates on a high-speed train in China. Co-travel times of infected persons and contacts range between 0-8 hours, and the average infection rate of contacts is found to be 0.32%. It is further found that closer proximity to infected cases and longer co-travel times are associated with higher risks of infection.
- Shen et al. (2020) analyse the infection rate on two buses undertaking a 100 minute round trip to and from common origin and destination locations in China. In the bus with one infected source patient, approximately 35% of passengers are infected. On the second bus with no infected source, no infections are recorded. The buses both had recirculating air systems, and further analyses of proximity of infected persons to the index case show that airborne rather than direct contact transmission was the likely mode of infection.
- Luo et al. (2020) analyse infection rates of passengers exposed to an index case on two bus trips in China, of 2.5 hour and 1 hour durations, respectively. On the first trip, approximately 16% of passengers (8 out of 49 passengers) are infected, while on the second trip, approximately 17% are infected (2 out of 12 passengers). On both bus trips, windows remained closed and a ventilation system was in operation; the location of secondary infections suggests that transmission was likely a result of airborne aerosols.

Along with the empirical evidence, theoretical studies have also been undertaken to predict SARS-CoV-2 infection rates on public transport. The most common approaches include application of the compartmental epidemiological model frameworks, and the Wells-Riley model specifically for the possibility of airborne transmission^2^. In the compartmental models, the population is assigned to different compartments, with the base model consisting of susceptible-infectious-removed (SIR) compartments (Kermack and McKendrick 1927; Brauer and Castillo-Chavez 2001). The objective is to estimate the reproduction number *R*, which is defined as the number of secondary infections caused by one primary infected case (Brauer and Castillo-Chavez 2001).

The main limitation of the theoretical studies is the application of assumptions for the input variables. The SIR and Wells-Riley based methods require input of the number of infected persons within the community and the infection transmission rate between two persons. The SIR model further requires estimation of the incubation periods and recovery characteristics of infected persons. However, these quantities are difficult to estimate, particularly given the dynamic nature of the pandemic and unknown transmission characteristics of new variants. The findings of the models can only at best demonstrate that transmission is possible on public transport; the calculated transmission rates depend heavily on assumptions of infection dynamics within the population, alongside assumptions of the demand and operational characteristics of public transport.

Mo et al. (2021) use a susceptible-exposed-infectious-removed (SEIR) model coupled with an encounter network model of contacts to estimate infection rates on the Singapore bus network using demand data for regular operations in 2014. The authors report that to reduce *R* below 1 (i.e. exponential decay in infections), interventions would be required, including: demand restrictions on the public transport network, partial closure of the network focusing on busy routes, and other public health interventions such as contact tracing and quarantine, wearing of PPE, and vaccination. The UK Rail Safety and Standards Board (RSSB) quantify the infection risk for an average passenger journey on UK rail using LEGION pedestrian modelling software (Hunt 2020). A toy network comprising 3 stations is used; passenger demand levels equate to one train carriage of passengers waiting at the platform, and passengers are assumed to occupy every second seat on the train. It should be noted that the study was published in August 2020, and so infection parameters were based on disease dynamics at that time. The RSSB acknowledges that the previously reported infection risk of 1 in 11,000 journeys or 0.009% per journey is likely to increase with the introduction of new variants. Dai and Zhao (2020) apply the Wells-Riley model to quantify the relationship between infection rates and ventilation rates on buses, among other enclosed spaces. Higher ventilation rates are found to be effective in reducing infection rates; moreover, if masks are worn, the ventilation rate can be reduced to 25% of regular levels to achieve the same rate of infection (1%) in cases where masks are not worn.

The key parameters influencing transmission, namely exposure distance to an infected case and contact duration, vary across the empirical and theoretical studies reviewed so far. The maximum distances for infection vary from 3m to 6m for straight line distances, up to entire enclosed vehicle spaces (Hunt 2020; Dai and Zhao 2020; Shen et al. 2020; Luo et al. 2020; Hu et al. 2021). The minimum values for exposure time range from zero to 15 minutes (Hunt 2020; Dai and Zhao 2020; Shen et al. 2020; Luo et al. 2020; Hu et al. 2021). In terms of more general recommendations on transmission distances, the World Health Organisation currently advises to maintain a minimum 1m separation distance between persons regardless of exposure duration (World Health Organisation 2021). In the UK, the current governmental guidance for travel on public transport is a 2m separation distance but if this cannot be achieved, a minimum 1m separation distance is advised (Department for Transport 2021). Jones et al. (2020) perform a review of the physical distancing literature and argue that the conventional 1-2m minimum recommended separation distances are based on dated studies which focus on large droplet transmission only. In more recent studies, the authors find that droplets can spread up to 6-8m, and when considering airborne transmission, the virus may remain viable in enclosed spaces at greater separation distances. Though the empirical and theoretical studies reviewed vary widely in terms of assumptions of virus dynamics, demand, and operational characteristics, we can infer that SARS-CoV-2 is more likely than not to be transmissible on public transport.

### Early policy recommendations

In the absence of population immunity via vaccination, social distancing remains the preferred control strategy to inhibit the spread of the SARS-CoV-2 virus. Social distancing policies typically involve the stipulation of a minimum separation distance that is recommended to be kept between persons in public areas, and this also explicitly applies to the use of public transport networks.

In their analysis of the impact of Covid-19 on over 100 international public transport operators, the TSC reports that most transit operators have adopted social distancing policies as mandated by government guidance, regulations and/or laws (Transport Strategy Centre 2020). On trains, a selection of operators have adopted maximum capacity limits ranging from 1.3 to 2.0 passengers per square metre, and across all operators questioned, railways are planning for 20-50% utilisation of maximum capacity. Bus operators have typically specified maximum limits of 10-20 passengers per bus. On latest government advice, some operators have relaxed these figures, adopting two thirds maximum capacity limits or 1 metre minimum separation distances, given that passengers wear masks. Nevertheless, it seems difficult to maintain these restrictions after the peak of the pandemic without further operational and demand management interventions (Coppola and Fabiis 2021).

A limited number of academic studies and review articles have been recently published to highlight potential measures that operators could implement to manage their networks during the pandemic. In their review article, Tirachini and Cats (2020) present a wide range of measures to enforce social distancing in public transport, with a focus on demand management:

- On demand services
- Reserved slot booking
- Travel permitted for specific groups only e.g. essential workers
- Pricing management (also suggested by Oum and Wang 2020)
- Peak spreading via societal changes in work, education, leisure timing
- Peak spreading via dissemination of crowding information to passengers (at stations, online, phone apps)
- Rail crowding management at station entrances, within station passageways, and at platforms
- Bus crowding management: dedicated bus lanes, headway management
- Updating service frequencies to new demand levels
- Changes in service patterns: altered stopping patterns, short turning (also suggested by Gkiotsalitis and Cats 2020b)

According to the TSC report surveying over 100 public transport operators (Transport Strategy Centre 2020), items (b), (d), (f), (g) and (i) in the list above have been already adopted by operators. Gkiotsalitis and Cats (2020a) use a mixed integer quadratic programming model to simulate and recommend the optimal frequency of services under different social distancing scenarios, while Oum and Wang (2020) apply conventional economic traffic congestion theory to advise on optimal lockdown periods and the potential for financial penalties to manage demand.

In addition to measures related to achieving social distancing, operators have also adopted other non-pharmaceutical measures including: mandatory wearing of masks, temperature screening, contact tracing, sanitation of exposed surfaces, and improvements in ventilation (Transport Strategy Centre 2020).

A number of recent empirical academic studies have investigated the effectiveness of social distancing and other non-pharmaceutical interventions in controlling virus spread (McGrail et al. 2020; Islam et al. 2020; Kraemer et al. 2020; Liu et al. 2021). Other studies have analysed the impact of restrictions on the reproduction number (*R*) via epidemiological compartmental models (Lai et al. 2020; Davies et al. 2020; Flaxman et al. 2020). The general consensus is that social distancing measures are effective in inhibiting virus transmission. It should be noted that most studies analyse cities where a number of concurrent measures have been adopted, and as a result, it is difficult to disentangle the impact of each measure alone. Other typical limitations of the studies include potential discrepancies in the degree of compliance with interventions, and potential discrepancies in virus testing results.

## Theoretical insights: The economics of social distancing

Section 2 has reviewed a range of early empirical findings, and we infer that some sort of supply-side re-optimisation of public transport services is inevitable during the pandemic. *Supply* has several dimensions in public transport: we focus on what service frequency and vehicle size are provided (together referred to as *capacity*) and what monetary fare passengers have to pay. This section revisits the economic literature of public transport supply, but the discussion emphasises that non-economics factors may also have to be taken into account in practical decision making.

### The optimal occupancy rate

What is the optimal occupancy rate during a pandemic? This question is very difficult to answer without reliable information on (i) the impact of physical proximity on the risk of contagion, given the travel distance and vehicle characteristics (e.g. the type of air ventilation), and (ii) the social cost of actually spreading the virus, including a series of consequences ranging between health care expenditures and the ethically highly sensitive valuation of human life. Under regular conditions, the literature suggests that the optimal occupancy rate is a derived quantity: operators optimise the available capacity and the level of demand through price and quantity controls simultaneously, and the resulting demand-to-capacity ratio determines the vehicle load in optimum.

Numerous studies have derived analytical formulae of the optimal occupancy rate, and illustrated its properties in numerical simulations. When the model of a representative origin-destination pair is considered, earlier studies show that the optimal occupancy rate is a relatively stable function of total travel demand (Jara-Díaz and Gschwender 2003). Even if density economies in operator costs are taken into account, vehicle load is just mildly decreasing in ridership (Hörcher and Graham 2018; Börjesson et al. 2019). On the other hand, when multiple line sections are served by the same capacity and demand fluctuates between these sections, it is unavoidable that certain segments of the public transport line are more crowded than the others. Depending on the magnitude of demand imbalances, very dense crowding may well be optimal from a social welfare point of view, at least for short time periods and in the main bottleneck of the line (Hörcher and Graham 2018).

Can we adopt the methodology of this literature to infer optimal supply strategies during the pandemic? In fact, most consequences of an infectious disease can be associated with well-known user-, operator-, and external costs. First of all, the (perceived) user cost of crowding is expected to be substantially higher due to potential infections. Passengers adjust their travel habits to their beliefs about the probability of getting infected and its impact on their life, including quarantine restrictions and potentially serious health degradation. Note that the infection risk is a *consumption externality*: the virus is transmitted from one user to another, and because this user cost increases with vehicle occupancy, the marginal traveller imposes an external cost on fellow users that is expected to be much higher than the regular inconvenience of crowding. Additional externalities might have to be taken into account by the welfare maximising operator because passengers who get infected on public transport could later on spread the virus as part of subsequent activities. Therefore, not only public transport users bear the full cost of potential contagion. To date, neither the exact infection probabilities, nor the user valuation of the crowding experience have been reliably quantified at the present stage of the crisis.^3^

Operator costs are also affected by Covid-19. For example, drivers and other staff members are more intensively exposed to health risks compared to regular passengers. As a consequence, many operators face staff shortages, and increased expenditures on protective equipment. These factors are likely to increase the cost of capacity provision, primarily due to the high cost of labour in hazardous jobs, cleaning, and various precautionary measures.

The literature proposes that public transport capacity (i.e. frequency and vehicle size) should be increased as long as the resulting marginal user cost reduction is greater than the marginal operator cost. The challenging part of re-optimising public transport supply and deriving the optimal occupancy rate is the calibration of the user, operator and external costs discussed above. Even though precise estimates are currently not available, both user and operator cost functions are expected to be steeper in the pandemic scenario compared to regular operating conditions. The marginal user gains from service frequency and vehicle size would be higher than usual, while the marginal operational costs would also be more substantial. Gkiotsalitis and Cats (2020b) discuss a series of additional supply-side measures to be adapted under the pandemic crisis, including short-turning, overlapping lines, vehicle holding and speed control.

When it comes to social welfare maximising pricing, theory suggests that the optimal fare should be set equal to the marginal non-personal cost of travelling (Small and Verhoef 2007; Pels and Verhoef 2007), which is often dominated by the marginal external crowding cost (Hörcher and Graham 2018; Börjesson et al. 2019). As noted above, we consider the risk of infection as an externality. Thus, for a given occupancy rate, the optimal financial (dis)incentive is expected to be stronger than usual. However, if the equilibrium occupancy rate is lower than usual, then we cannot claim with certainty that public transport fares should be higher during the pandemic. This implies that the optimal capacity and pricing adjustment are both ambiguous, and therefore we cannot speculate about the optimal occupancy rate without reliable estimates of key input parameters. Differentiated pricing as a demand management tool is further investigated in Section 4.2.

We can derive one important conclusion from the discussion above. The determination of the optimal occupancy rate is not merely an infectious disease control problem *per se*. In the absence of infection probabilities and other input parameter estimates, the best (and many times the only thing) that public transport operators can do is to follow governmental guidelines and the most up-to-date regulation on social distancing.

### Demand management with occupancy restriction

Let us now revisit the public transport operator’s supply optimisation problem, assuming an exogenous upper bound on occupancy rates due to social distancing regulations. The exogenously fixed occupancy rate was a usual assumption of public transport models prior to the widespread use of crowding cost functions. Many authors, including Jansson (1980); Chang and Schonfeld (1991); Small (2004) and Basso and Jara-Díaz (2012) derived the optimal vehicle size as the ratio of aggregate demand and service frequency, assuming (in the absence of crowding costs) that operators always utilise the entire vehicle capacity. If crowding costs are taken into account but social distancing imposes an additional constraint on the occupancy rate, then three possible outcomes are anticipated:

1. The optimal occupancy rate may remain under the social distancing threshold, in which case capacity optimisation should follow the methodology summarised in Section 3.1.
2. If the vehicle occupancy restriction becomes binding, then crowding costs are no longer relevant, and vehicle size should be set such that the occupancy rate remains just under its threshold level. Thus the findings of early public transport models mentioned in the previous paragraph could become relevant again.
3. Further restrictions might be applicable if frequency and/or vehicle size are also constrained, for example due to technological limitations. In this case additional demand management efforts seem inevitable.

A detailed numerical calculation for a specific case study area is out of the scope of this paper. However, due to the high cost of crowding in a pandemic situation, the authors’ speculative view is that the optimal frequency and vehicle size are higher than usual. However, capacity expansion may be hindered by the technological limitations, indicated in item 3, that can be prevalent in many large cities, especially in older European cities where both bus and urban rail capacities are restricted by land use and technological constraints.

This raises the importance of additional demand management efforts when social distancing policies must be introduced. Assume now that both the service frequency and vehicle size are fixed in the short run, and the operator is facing temporal demand shocks when the unregulated ridership is likely to exceed the critical occupancy rate. In addition, travelling causes regular social costs including crowding externalities. While buses and trains can normally be used up until their physical capacities, the imposition of social distancing rules implies that passengers should not be allowed to board the vehicles at some point, even if it would be possible, physically, to board. The economic efficiency criteria of demand management with an upper bound on vehicle occupancy rates are summarised in the Appendix of this paper.

Economic efficiency is not the only allocational criterion in society, however. Passengers differ in many respect beyond their willingness to pay to travel, and policy makers might wish to prioritise travellers according to other characteristics such as income or residential location. Further discrimination can be made in a pandemic scenario according to trip purpose. For example, key workers’ access to mobility is a crucial requirement in the crisis, for obvious reasons. More generally, commuting to work might enjoy higher priority when it comes to restarting urban economies, while households should receive additional incentives to perform shopping and other leisure activities at not too distant locations.

### Demand management in networks

**Fig. 1:**
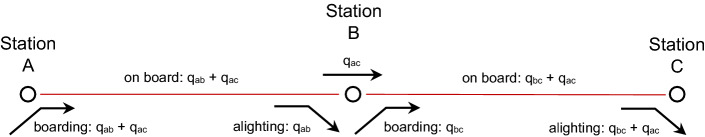
On-board ridership and boarding and alighting rates in a simple unidirectional network of three stations

**Fig. 2:**
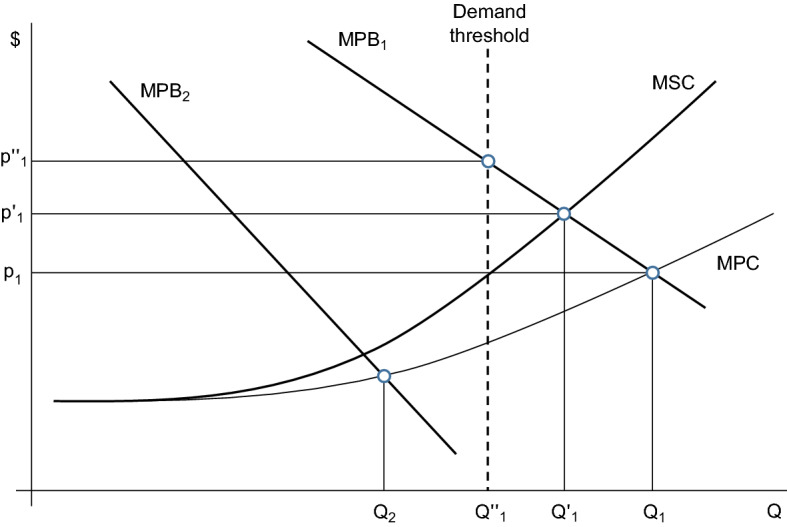
The purpose of demand management with and without an explicit occupancy constraint and varying demand conditions

Demand management to enforce social distancing becomes more complex when multiple origin-destination (OD) pairs are served by the same line capacity. Consider a simple public transport line between stations A, B and C, serving three origin-destination pairs with demand $q_{ab}$, $q_{bc}$ and $q_{ac}$. This simple network layout is depicted in Figure 1. Social distancing now requires that $q_{ab}+q_{ac}$ and $q_{bc}+q_{ac}$ *both* remain under the critical vehicle occupancy rate. These two sums could be considered as two demand curves in the market representation of Figure 2 in the Appendix. However, as AC passengers are present on both network segments, the two demand curves are interlinked, and demand management on the first line section would imply a shift in the demand function of the second section. Thus, demand measures have to be designed by considering two vehicle occupancy restrictions as well as the simultaneous dependency of the ridership on different OD pairs.

In networks, multiple origin-destination pairs compete for the same capacity on shared sections, and therefore demand management involves allocation *within* as well as *between* OD pairs. Assume for example that the social distancing constraint is binding on Section BC, and therefore either $q_{ac}$ or $q_{bc}$, or both, would have to be controlled to some extent. The optimal control strategy will depend on whether AC or BC trips are valued higher, according to economic or other criteria such as equity. One option is to prioritise long-distance trips, as these passengers have fewer alternatives for commuting, and therefore their willingness to pay for travelling might be higher. However, this economic rationale raises spatial equity concerns as the burden of complying with social distancing rules would be put on BC passengers disproportionately. Political economy considerations might also come into play as long-distance travellers are often residents of different electoral jurisdictions, while local decision makers might prefer prioritising local urban residents. In large public transport networks dozens or even hundreds of OD pairs, including transfer passengers, may share the most densely used line sections, which makes the control problem increasingly complex.

The efficiency of the solution to the network-level allocation also depends on whether it is feasible to discriminate between OD groups, i.e. whether the operator can prioritise certain origin-destination pairs against others. Most of the existing demand management methods imply either station or section based processes, meaning that demand is restricted based on the location of boarding, or based on the line sections used. None of these approaches can ensure OD-based differentiation. In other words, $q_{ac}$ cannot be restricted with a station-based approach, as AB and AC passengers enter the network at station A together, and the operator might not be able to identify and discriminate the two groups. Section-based control of $q_{ac}$ is also unfeasible, as $q_{bc}$ is also part of the on-board train flow between B and C. Thus, station and section based interventions, which are easier to implement physically, are more restrictive from an efficiency point of view than OD-level differentiation.

## Practical demand management methods

The key message of Section 3 is that due to the lack of reliable empirical estimates of key cost parameters, it is difficult to find the optimal occupancy rate during Covid-19. It is more likely that public transport operators (will) need to follow external social distancing rules in daily operations. The third objective of this paper is to investigate what demand management methods might be available to comply with such rules, how efficient they are according to various criteria, and what practical difficulties may hinder their implementation.

Throughout this section we assume that social distancing can be ensured by controlling the flow of passengers into the public transport system. The system boundary is at station entrances and exits (e.g. fare gantries) in case of rail services and at the doors of vehicles in case of buses. Based on the frequency and interior capacity of vehicles, one can derive the upper bound of ridership that can be served without exceeding the critical occupancy rate. Naturally, if headways are irregular and the distribution of passengers is uneven within the vehicles and on platforms, then social distancing requires that the planned occupancy rate remains lower than the external regulation. We assume that operators have sufficient knowledge and data to determine the necessary correction factor, and thus the section’s focus is on the regulation of system inflows.

### Inflow control with queueing

Inflow control implemented with a queueing system is the simplest demand management method which is already applied in several large urban rail networks, primarily to avoid station overcrowding and the associated safety risk. Inflow control is implemented by physically restricting the flow of passengers entering the metro station, and storing the excess flow in a physical queueing system. In large networks inflow controls might have to be applied at numerous stations to achieve the demand management objective. Thus, the optimisation of the inflow control policy includes the determination of (i) which stations should be controlled, and (ii) the upper bound of inflows at each station.

Conceptually, the idea of inflow control is rooted in the literature of highway ramp metering, which is a closely related problem in road traffic management. The literature of highway ramp metering dates back to the 1960’s (see e.g. May 1965; Wattleworth 1967); its primary purpose is to keep traffic flows under a pre-estimated threshold by limiting the number of vehicles entering the highway at on-ramps. In case of road traffic, this critical flow is determined by the point where the road section reaches its capacity, and additional traffic would deteriorate its total throughput due to hypercongestion (Daganzo 1997). The control problem is not trivial, because inflows can be restricted at any on-ramp upstream to the active bottleneck on the highway, and new (downstream) bottlenecks can also emerge as a result of the intervention. Users might re-optimise their travel after the interventions, thus increasing the complexity of this two-level control problem (Yang et al. 1994).

The literature of ramp metering is hallmarked by repeated efforts to improve the optimisation heuristics based on a known, time dependent demand matrix of each on-off ramp pair along the highway. The objective function of the problem is normally to minimise the total time spent in the system, or minimise queueing delay at on-ramps subject to the critical flow restriction on the main highway. Lovell and Daganzo (2000) develop a general non-anticipative heuristic appropriately representing the temporal dynamics of the problem, i.e. that the effect of control measures at upstream stations is realised at the bottleneck with a time lag. They point out two important characteristics of inflow control, from a practical implementation point of view. First, they discuss the potential importance of whether inflows can be differentiated at a given origin by destination. This is impossible with traditional signalised ramp metering technology, but they hint that differentiation would make the system more efficient. Second, they note that “the question of where to store excess demand to a congested system can be very political”. Locally, extensive queueing at on-ramps may spill back to the urban road network, inducing external costs for residents, while on a system level politics might be involved in the determination of which on-ramps should be controlled the most. Very similar challenges might appear in a public transport application: queueing requires space, and controlling inflows at a limited number of stations can be perceived unfair.

Is a full time-dependent OD demand matrix required to optimise a ramp metering policy? This question is especially relevant in the context of social distancing in public transport, as demand patterns change regularly and often unpredictably during a pandemic scenario. Zhang and Levinson (2004) argue that OD demand data are hard to estimate, and do not even exist in reality, as demand levels are endogenous with respect to the supply-side interventions themselves. They develop a heuristic control logic based on simple intuition, which can be explained using the network layout in Figure 1. Assume again that social distancing would be violated on line section BC in the absence of intervention. The operator may limit inflows at Station A or B, or both. Zhang and Levinson (2004) show the general principle that inflows should be controlled as close to the bottleneck as possible, in this case at Station B only. The reason is that restrictions at Station A would affect the AB market as well, while they actually do not contribute to demand in the bottleneck. Based on this principle, they develop a heuristic to solve the inflow control problem. The only data requirement of their method is the share of off-ramp exit percentages, which is equivalent to the ratio $q_{ab}/(q_{ab}+q_{ac})$ in the present public transport application. They argue based on descriptive data analysis that off-ramp exit percentages are relatively stable over time, and therefore their algorithm requires substantially less data collection effort than the estimation of time dependent OD matrices.

Zhang and Levinson (2004) discuss some of the equity aspects of their general solution to ramp metering as well. They acknowledge that the most efficient metering regime is also the least spatially equitable one, as it implicitly minimises the number of metered on-ramps along the highway. They enlist a couple of practical remedies to relax the potential equity concerns. One of them is a maximum queue restriction combined with minimum/maximum metering rates: this is equivalent to setting an upper bound to the time that individual passengers would have to wait at entry stations. A similar result can be achieved by defining an increasing function for the unit cost of queueing time, which eliminates excess queueing in the policy optimisation process. In a follow-up paper Zhang and Levinson (2005) propose an advanced heuristic which distributes queueing costs among a predefined number of entry points in the most efficient way, thus balancing spatial equity and efficiency in the system.

The ramp metering literature has inspired several public transport applications. The benefits of boarding control have been recognised in the literature of operational control strategies,^4^ focusing mainly on headway regularity, bus bunching, and the associated degradation of user experience. Delgado et al. (2009, 2012) investigate boarding limitations in combination with more traditional bus holding strategies in a rolling horizon optimisation framework suitable for real-time operations. They show in a numerical simulation that if headways are short (under 10 minutes) and demand is close or above the physical vehicle capacity, then boarding control can achieve an extra 6.3% expected waiting time savings relative to the already substantial benefits of optimal bus holding. In addition, boarding control evens out bus occupancy rates, thus reducing the crowding inconvenience experienced by the average user, which might not be achieved even if headways are perfectly regular (Ceder 2001).

Passenger inflow control has gained more attention in recent years in the context of urban rail systems. The following studies showcase diversity in terms of the objective function of their control mechanisms:

- Guo et al. (2015) minimise the sum of queueing time and waiting on platforms subject to a station capacity constraint determined by safety regulation. Trains can be used up until physical capacity. Their solution method is particle swarm optimisation with a fixed OD demand matrix.
- Bueno-Cadena and Muñoz (2017) combine passenger trip times with operator costs stemming from energy consumption and minimise the resulting social cost function through three measures: speed control, train holding and boarding limits. They apply a standard numerical solver to optimise the model.
- Jiang et al. (2018) apply a queueing and waiting time based objective function similar to Guo et al. (2015), but the unit value of wait time increases exponentially, especially if the passenger fails to board more than two trains. Their primary motivation is also to avoid overcrowding in stations for safety reasons. The solution method is reinforcement learning, previously applied in traffic flow control (see Walraven et al. 2016).
- Shi et al. (2018) contribute to the literature by jointly optimising train timetables and station inflows to avoid platform overcrowding and minimise passenger wait time at station halls and on platforms. They propose an integer linear programming model and solve it with a hybrid heuristic based on a standard integer solver and local search.
- Zou et al. (2018) develop a feedback-based bottleneck elimination strategy to optimise inflow controls on a network level. They associate station inflows with section flows using a traffic assignment model, and establish a heuristic inflow control algorithm to eliminate the bottleneck(s) where the predicted demand exceeds the available capacity limit. This study lacks an explicitly defined objective function, but several practical considerations are built into the control algorithm. The heuristic itself is similar to the ramp metering method of Zhang and Levinson (2005) in the sense that they intend to control a given number of upstream stations directly preceding the bottleneck section.

A common limitation of the literature reviewed above is that they assume fixed (inelastic) OD demand matrices. This assumption is neither realistic nor helpful when the aim of inflow control is to reduce aggregate travel demand. With the user cost minimising objective one cannot differentiate the value of individual trips based on willingness to pay or any other criteria, and therefore the allocational performance of the demand management method cannot be evaluated either. This is a major limitation for social distancing applications.

Queueing might take a considerable share of the total travel time, and a bulk of empirical evidence proves that travel demand is sensitive with respect to trip duration (Wardman 2012). Practically speaking, if the queues are very long in front of the station entrance, some passengers may look for alternative means of transport, or reschedule their trips, or decide not to travel at all. Under the more realistic *elastic* demand assumption, queueing achieves an allocation of the available transport capacity based on passengers’ sensitivity with respect to travel time. Queueing prioritises those (i) for whom the trip delivers substantial personal benefits, and (ii) whose travel time valuation is relatively low. As these two characteristics might be inversely proportional (people with high value of time may find it more important to travel), the economic efficiency of this allocation method is questionable. In addition, the time lost in queues is a foregone resource for society, which is a huge disadvantage compared to other allocation methods such as pricing, in which case fare revenues can be recycled and utilised elsewhere within society. Nevertheless, queueing is generally considered as *fair* policy, because passengers at a given entry location have to spend the same amount of time in the first in, first out (FIFO) system. In addition, if low income groups have lower travel time valuation, then queueing is inefficient but progressive from a distributional point of view.

#### Practical applicability

Queueing systems are frequently used when entering crowded public venues such as museums, concert halls and tourist attractions. Passengers might see it inevitable that queueing is introduced when demand exceeds the capacity enabled by social distancing rules. The inefficiency of inflow control might not be visible for individuals in the short run, as the distribution of inflow rates set by the public transport operator at various entry stations is not known by users. This makes inflow metering with queueing in front of stations an evident solution to ensure the functioning of public transport under social distancing constraints.

It is important to note, however, that queueing might be a source of infection risk in itself. Queueing with sufficient physical distancing requires a lot of space which might not be available in or outside busy stations. Even if the required space is available, human assistance might be needed to ensure that potentially impatient passengers keep the safety distance at all times. Thus, the management of queueing systems during the pandemic would be more resource intensive than usual.

Lovell and Daganzo (2000) and subsequent authors have pointed out that the efficiency of inflow control is substantially higher if users can be differentiated by destination, or more importantly on the basis of whether they will travel through active bottleneck(s). Differentiation is hardly feasible in regular highway ramp metering. However, smart card technology in public transport provides ex-post information on the destination of travellers. This opens up the possibility of establishing multiple queues at entry stations depending on trip destination. Queues for OD pairs leading through bottlenecks are expected to be longer, but violations of the differentiated queueing systems could be identified and fined by cross-checking the entry gate data with the destination station in smart card data records. Again, the practical limitation of this idea is that destination-differentiated entry queues require even more space at entry stations.

### Differentiated pricing

Pricing in public transport is an often debated subject in the policy arena, as it is the main determinant of how affordable public transport is, and to what extent public budgets have to contribute to deficit financing through subsidies. The economics literature promotes the principle of marginal social cost pricing in public transport. Theory suggests that social welfare is maximised if the fare equals the gap between the marginal *social* cost and marginal *personal* cost of travelling (see Figure 2), in which case only trips with a non-negative net welfare effect will be realised.

Pricing techniques have advantageous theoretical properties in achieving both quantitative and allocative demand management goals simultaneously. Pricing enables that the personal cost of travelling can be set to any desired level between the unpriced equilibrium and the highest willingness to pay along the inverse demand curve. With advanced monetary transfer techniques even negative payments might be possible to incentivise travelling, in the form of a direct subsidy for public transport use. Pricing allocates the available capacity based on passengers’ willingness to pay for the service: assuming rational consumer behaviour, the sum of the monetary fare and non-pecuniary user costs form the lower bound of personal benefits among the actual travellers who opt for using the service in equilibrium. The main advantage of pricing as a demand management tool is that monetary payments remain within society, so that the amount by which the personal travel cost is raised can be later on redistributed among members of society, as opposed to the time lost in queues, for example.

The primary goal of the pricing literature has been to derive the net non-personal cost of the marginal trip in plausible models of public transport operations. This incremental welfare effect is determined by the following (sometimes off-setting) mechanisms.

1. Direct social costs and benefits without capacity adjustment
  Crowding disutility, as an externality (Tirachini 2013; Hörcher 2018).
  Delay costs during boarding and alighting (Jansson 1980; Oldfield and Bly 1988; Jara-Díaz and Gschwender 2003).
  Substitution with underpriced car use (Parry and Small 2009; Basso and Silva 2014).
  Wider economic benefits, including agglomeration economies (Venables 2007; Hörcher et al. 2020).
2. Additional welfare effect due to responsive capacity, i.e. adjustments in service frequency and vehicle size
  User cost savings, the Mohring effect (Mohring 1972, 1976).
  Marginal cost of public funds (Kleven and Kreiner 2006; Proost and Dender 2008).
  Density economies in operating costs (Anupriya et al. 2020b).

If an operator’s goal is to achieve social distancing with pricing, the regular supply optimisation problem has to be extended with an additional constraint on the equilibrium occupancy rate. This requirement adds a shadow price to the marginal social cost of travelling if the social distancing constraint is binding, and therefore the optimal fare is expected to be higher than its unconstrained equivalent. Eventually, the fare should raise the generalised price of travelling to the marginal willingness to pay at the demand threshold (see $d_1(Q''_1)$ if Figure 2). In other words, a critical precondition of enforcing an efficient allocation under social distancing with pricing is the availability of precise information on the inverse demand functions in all spatio-temporal markets of the network. Given that the demand function fluctuates over time while the demand threshold remains constant or non-binding, the optimal fare system might also have to be differentiated by time periods.

Another branch of the literature develops dynamic models of public transport demand in which travellers’ departure time choice is endogenous, but their desired arrival time is clustered in a narrow time window. Conceptually, these dynamic models resemble the traditional bottleneck problem of road traffic management and pricing (see recent reviews by Small 2015; Li et al. 2020). In public transport, the purpose of the dynamic fare is to replace the user cost of queueing with a payment. An optimised time-dependent fare schedule would essentially achieve the same temporal distribution of departures without queueing, by setting the fare equal to the monetary value of queueing time loss in the unpriced equilibrium (Huang 2000; Kraus and Yoshida 2002; Small and Verhoef 2007). Even though bottleneck models are normally governed by the physical capacity of the infrastructure, social distancing can be implemented assuming that an exogenous occupancy rate must not be exceeded in any line section, even if physical capacity would allow for it.

#### Practical applicability

Achieving social distancing with pricing tools seems to be a challenging task from a methodological point of view. Most of the theoretical models in the literature rely on explicit demand functions, and in case of dynamic models also on the distribution of desired departure or arrival times. Such demand information would also have to be disaggregate both spatially and temporally to control demand in a large network continuously.

The road pricing literature offers several algorithms to solve the optimal pricing problem without explicit demand functions. The intuitive idea behind trial-and-error based pricing schemes comes from Downs (1993) and Vickrey (1993), which is then formalised by Li (2002) in a bisection toll adjustment method. Yang et al. (2004), Han and Yang (2009) and later on Wang and Yang (2012) enhance this approach for a general road network, and prove its global convergence properties. Guo et al. (2016) develop a practical method to restrain demand under a predetermined flow level, which may not be equivalent to the welfare maximising flow; this resembles the case of social distancing. Guo et al. (2020) extend this line of research to a bimodal operational scheme in which bus fares as well frequencies are responsive on a day-to-day basis. Purely public transport related applications of the trial-and-error pricing methods are rare in the literature. The study of Wang et al. (2018) is an exception, but they adopt this iterative approach for a very specific demand management task: to redirect passengers from busy central stations to neighbouring ones by adopting as little fare differentials as necessarily required.

A common property of the aforementioned pricing methods is that they make two key assumptions: (1) The unknown demand function is stable over time, and even if demand is in disequilibrium temporarily, it converges to a steady state eventually, and (2) the speed at which travellers react to price adjustments is known, and in principle it happens instantaneously. Unfortunately, the travel demand functions might not remain stable on a day-to-day basis in a pandemic scenario. Recent experience suggests that in parallel with rapidly changing disease control rules and fluctuating economic performance, travel demand may oscillate unpredictably on a day-to-day basis, even in the absence of supply-side interventions. Also, not every traveller is informed about price adjustments instantaneously,^5^ and even if they are, their behavioural reaction may take longer than expected. Relying on an iterative pricing tool under such circumstances can be hazardous, because the realised demand may easily exceed the desired occupancy threshold on certain days, or the demand control may also be over-insured and therefore too restrictive.

The social acceptance and political implementation of peak pricing is another major challenge of practical applicability. Demand management with pricing involves large financial transfers from public transport users to government, which is generally unpopular. Low income travellers might be especially adversely affected by the policy during a period of economic downturn. The aversion of the public can be limited if the channels of redistribution are transparent. Changes in travel behaviour can be achieved by rewarding, i.e. negative pricing, which is indeed much better perceived in the public opinion (Rouwendal et al. 2012). The downside of extensive reward schemes is the pressure it puts on the public budget at a time when transport operators are already severely financially constrained, and rewarding off-peak travellers might be unfair against those residents/voters who do not travel at all. While some empirical evidence on the success of rewarding does exist in the context of road use (Knockaert et al. 2012), experience in public transport, as documented by Anupriya et al. (2020a), suggests that the behavioural response might be slower, less intensive, and more expensive than what social distancing during the pandemic would require. On the theoretical front, promising new findings by Tang et al. (2020a, b) indicate that the integration of fare-reward schemes with non-rewarding uniform fares may achieve demand management goals revenue neutrally.

Due to the practical limitations above, it is more plausible that time-dependent dynamic pricing could play a complementary role beside physical inflow control, i.e. queueing in front of stations. As soon as such inflow controls are in place, queue lengths are immediately observable and the corresponding user costs are also easy to estimate. Replacing queueing costs with monetary payments, in line with the original dynamic pricing theory of traffic bottlenecks (see Small 2015), seems to be a more manageable goal compared to a purely pricing based demand management policy for social distancing.

### Advance booking and slot rationing

The disbenefits of queueing, especially the cost of time loss and its uncertainty, have been recognised in many industries where capacity allocation is organised through reservation systems. Within the transport sector, reservation is mandatory for many low frequency public transport services such as long-distance rail or air, or ferries, because running out of capacity in a given time slot would otherwise cause intolerable user costs. The potential implementation of advance booking on highways has been raised repeatedly by Wong (1997); Koolstra (1999); De Feijter et al. (2004) and Edara and Teodorović (2008) for unique road sections, and by Zhao et al. (2010) for a cordon-based downtown area. More recently, Lamotte et al. (2017) revisited this idea in the context of autonomous vehicles, and Menelaou et al. (2018) propose a reservation based demand management method for entire urban road networks. Interestingly, urban public transport related applications are sparse in the literature.

The basic rationale behind slot reservation is to avoid unproductive time loss in queues. Advance booking is a simple quantity control method which prevents more trip plans being made than what is actually feasible in a given time period. Replacing queueing with reservations offers several benefits for both users and the operator. Beside the regained time in the absence of queueing, advance booking makes the trip duration more reliable, saving additional schedule delay costs due to early or late arrivals. This user benefit may be substantial in a morning commuting scenario (see, e.g., Peer et al. 2012). Additionally, advance booking provides important benefits for the operator as well, in the form of much more predictable demand patterns. Prior information on unexpected demand shocks might be extremely valuable for service planning and management, even if the reservation system is not meant to eliminate queues entirely. The advance reservation requirement enables the operator to explicitly reject travel requests before passengers arrive at their trip origin. Whilst this is certainly not a desirable outcome *per se*, advance rejection may still cause less harm and annoyance for the passenger than modifying the travel plan after she spent a considerable amount of time in queues.

Ideal demand management under the pandemic serves quantitative as well as allocative goals simultaneously. It is clear that advance booking achieves the quantitative goal very well if no more time slots are made available than what social distancing rules enable. The allocative efficiency of regular reservation tools based on a FIFO principle is more questionable, however. In this case capacity is allocated to those users who make their trip decisions earlier, and last-minute requests are more likely to be rejected. There is no clear connection between the time of booking and the value of trips for society,^6^ and therefore the efficiency of the FIFO reservation policy is ambiguous.

Operators might be able to improve the allocational efficiency of the reservation system by setting the price of advance booking in such a way that spare capacity remains available until the end of the booking horizon (i.e. just before the train or bus departure). This strategy would seemingly resemble the pricing strategy of airlines, where fares normally increase in function of the time of booking. The literature suggests that profit maximising firms have two distinct motivations behind this strategy: (i) to handle unexpected demand shocks, and (ii) to apply intertemporal price discrimination, exploiting the fact that last-minute travellers’ willingness to pay tends to be higher than the early bookers’ reservation price (McAfee and Te Velde 2006; Williams 2020). With a social welfare oriented objective, there is no reason to apply price discrimination, but the ability to handle stochastic demand variations might be important, especially in a pandemic environment. Reaction to stochastic demand means that the operator (1) follows the evolution of bookings for each capacity slot over the booking horizon, (2) models the regular pattern of booking requests, and (3) adjusts the price of reservations upwards (downwards) if the booking pattern exceeds (subsides) the regular pattern over time (see the equivalent process described by Williams 2020, in the context of airline pricing). This way the main challenge of regular pricing methods discussed in Section 4.2, namely the difficulty of *ex-ante* demand function estimation, can be overcome very effectively.

Finally, a slot reservation system enables the operator to pre-assign capacity to specific groups of travellers. This might be important in order to ensure that key workers can reach hospitals, care homes, schools or other destinations safely and in compliance with social distancing rules. The reservation system is also suitable to give priority to disadvantaged travellers, and adopt price discrimination based on social equity considerations.

#### Practical applicability

The implementation of an online travel slot reservation system via smartphone applications and other electronic channels is feasible from a technological point of view.^7^ Alternative means of advance booking must remain available for disabled passengers, those who experience technical failures on their devices temporarily, and those who do not have access to such devices. The reservation and ticketing system must also comply with the relevant privacy regulations.

The key prerequisite of the successful implementation of a network-wide reservation system is that both passenger and train movements remain punctual. Naturally, if passengers arrive earlier than the pre-booked entry time, they have to wait in front of the station which requires sufficient buffer space locally. Late arrivals cannot be corrected this way, and therefore the system has to be prepared for minor adjustments in individual itineraries. The scheduling problem is even more pronounced in large multimodal networks where late passenger arrivals at entry stations might be caused by unreliability or disruptions on feeder services. If the available capacities are fully booked in a given period of time, disruptions might have a cascading effect throughout the network, as entry queues caused by the delay cannot be reduced rapidly. Nevertheless, the fact that the operator has reliable information on future demand can make disruption management with the use of extra capacity and optimised diversions more effective than usual.

### Advanced quantity control techniques

The final group of demand management methods discussed in this paper are also direct quantity control tools with advance booking, but their slot allocation processes are based on bidding mechanisms instead of a FIFO rule. We first discuss permit auctions, followed by an extension to tradeable permit schemes.

An alternative way in which capacity reservations can be organised is an auction system where potential travellers bid for the available capacity. In order for an auction mechanism to reach efficient capacity allocation, an iterative process has to be implemented, primarily to allow the winning bids to approach the optimal market price, and leave sufficient consumer surplus with travellers. In a digital environment, the bidders do not need to be the users themselves; their preferences can be represented by an automated bidding logic, and thus several hundreds of auction iterations can be performed virtually within reasonable computing time (Iwanowski et al. 2003).

Once again, we find more road use related applications in the literature. In a recent contribution, Su and Park (2015) develop a highly granular agent-based simulation tool in which travellers’ value of time and preferred arrival time are unique. Their bidding logic performs a blind and greedy search among the available travel time intervals on a highway, with varying bid levels, in a series of consecutive iterations. They show the convergence of the bidding process and its effectiveness in guaranteeing congestion-free travel on the simulated highway section. This auction algorithm has not yet been adapted for public transport.

After an auction or a direct initial allocation of travel permits, their holders may also be allowed to resell their right to travel on secondary markets, thus implementing a tradeable travel permit scheme.^8^ The concept of tradeable credit mechanisms as an alternative to congestion pricing has been present in the road traffic management literature since Verhoef et al. (1997) and Goddard (1997), and in-depth reviews of this emerging literature are provided by Fan and Jiang (2013), Grant-Muller and Xu (2014) and Dogterom et al. (2017).

The main benefit of a tradeable permit scheme, in comparison with pure pricing techniques, is on the equity and social acceptance side. The essence of such schemes is that travel credits are distributed among all residents according to a predetermined rule, and then those who actually intend to travel regularly must buy additional credits from those who do not. Thus, the scheme achieves an efficient allocation of the available capacity, but those who would be priced off public transport when capacity is limited receive a direct monetary payment. Potential users with low or zero willingness to pay can earn a net income from the scheme. It is well known that the social acceptance of usage-based transport pricing depends heavily on how the revenues are redistributed (Parry and Bento 2001) – the tradeable permit scheme is in fact an auction where revenues are immediately redistributed to non-users, thus making all groups of society interested in the implementation of the policy, and avoiding large monetary transfers to the government. The initial allocation of quotas can be uniform among the population, or non-uniform according to the regulator’s distributional objective. Brands et al. (2020) raise a word of caution: The initial allocation of permits is also susceptible to corruption. This threat could make the social acceptance of permit schemes less trivial in many emerging economies.

#### Practical applicability

Even though the theoretical advantages of capacity auctions and tradeable credit schemes are consensual in the literature, their implementation with the aim of achieving social distancing in public transport may be challenging. The challenges stem from the spatially and temporally unstable nature of demand, which requires that capacity, which is perishable, has to be defined individually for each line section and time period. In broad terms, this highly disaggregate product can be auctioned and traded in multiple ways:

(A) Each spatio-temporial block of capacity has to be associated with a unique set of permits, which are then auctioned/traded prior to its period of validity, individually.
(B.1) Travel credits are used as a dedicated currency for travelling with their own market value, and the operator or regulator determines how many credits have to be paid for using a spatio-temporal segment of capacity, depending on how stringent the capacity constraint is. Users exchange the credits directly, as part of an online bargaining process or brokerage. The volume of credits is constant.
(B.2) Travel credits are used as tokens with a time-varying credit charge function, but the credits have to be bought or sold from or to a centralised *bank*, which controls their price. Thus, the volume of credits may vary over time.
(C) Travel credits are associated with a given amount of transport consumption. In road transport, this is often measured by vehicle miles travelled (Verhoef et al. 1997; Yang and Wang 2011), the days of week when access is unlimited (Goddard 1997), or the number of individual trips to be taken (Fiorello et al. 2010). Restrictions may apply though on the geographical area and time of day that the credit can be used.

Options B.1, B.2 and C offer several practical benefits in general transport capacity allocation problems, but their usefulness for the specific case of social distancing in public transport appears to be limited. To implement B.1 and B.2, the same amount of demand-side information would be required when setting the credit price of capacity segments as what differentiated pricing requires (see Section 4.2), to ensure that demand never exceeds the critical occupancy rate. This version of the tradeable credit scheme is in fact equivalent to a revenue neutral monetary surcharge-reward scheme proposed by Kalmanje and Kockelman (2004) for road pricing, and more recently adapted to public transport by Tang et al. (2020b). Moreover, Bao et al. (2019) show that option B.1 might lead to an unstable equilibrium in the standard bottleneck model, and therefore the welfare gain it provides is also uncertain.

In option C, permits would allow travelling a predetermined distance in the public transport system. Given that demand can be heavily imbalanced both spatially and temporally, controlling the total passenger miles within the system does not guarantee that social distancing is not violated anywhere and anytime.

This leaves us with option A, if the goal is to keep vehicle occupancy rates below an exogenous level. This can be considered as the public transport equivalent of the road demand management scheme proposed by Akamatsu and Wada (2017). Verhoef et al. (1997), Yang and Wang (2011) as well as Fan and Jiang (2013) raise concerns about the practical implementation of this approach, as “the [roadway] network may produce a tremendous number of distinct permits for every [roadway] link, at each time interval”, which makes trading “practically inconceivable” (Fan and Jiang 2013). Akamatsu and Wada (2017) defend their position by proposing that “the implementation of these [time-space specific tradeable permits] would become feasible with advanced vehicles in which an agent software is installed to automatically trade permits based on users’ preferences”. The behavioural and travel demand implications of automated permit trading has not yet been shown in the literature, to the best of our knowledge.

Despite considerable efforts in the past 25 years aimed at understanding the theoretical properties and economic performance of travel demand management with tradeable permits, practical implementations are rarely reported in the literature. Brands et al. (2020) document the first such experiment we are aware of, in which a small sample of participants tested a virtual parking permit application. Off-the-shelf methods for tradeable permit schemes are not yet available for implementation in public transport.

## Conclusions

The ongoing Covid-19 pandemic imposes unprecedented challenges on the public transport industry. Demand and fare revenues have plunged to unprecedentedly low levels; furthermore, mixed messages have been disseminated to travellers on the risk of infections and safety in public transport in general. It is likely that urban economies will not be able to return to their pre-Covid level of productivity without efficient mass mobility. Due to the unprecedented nature of the challenge, public transport operators must respond with innovative and often unexplored solutions to the crisis, building on emerging technologies.

After reviewing the existing empirical evidence and the theoretical principles of network-level demand management, the paper has discussed five potential approaches to social distancing in public transport: (i) inflow control with queueing, (ii) time and space dependent pricing, (iii) capacity reservation with advance booking, (iv) slot auctioning, and (v) tradeable travel permit schemes. In general, none of these methods offers a simple and efficient implementation path towards controlling occupancy rates if implemented in isolation.

- Queueing might be easily understood and accepted by passengers in a pandemic scenario, but it generates substantial efficiency losses due to the time lost unproductively in queues.
- Dynamic pricing approaches realise demand management more efficiently, as fare revenues can be recycled within society, but their implementation requires costly data inputs to estimate the shape of (dynamic) demand functions *a priori*. The social acceptance of peak pricing during the pandemic is also uncertain.
- Capacity reservation offers numerous advantages in service planning and operations, and it is also capable of eliminating queueing at network entry points. However, a network-level slot reservation system requires highly predictable travel times, as passengers may lose their slots due to delays caused by disruptions or headway deviations. Also, capacity allocation is inefficient if a simple first come first served (FCFS) reservation rule is implemented.
- Slot auctioning is an attractive method to charge public transport capacity at an efficient price without additional demand data. However, to achieve this, a large number of bidding iterations must be calculated, and this is only feasible in a high performance computer aided environment.
- Tradeable travel permit schemes provide the added benefit that demand management is revenue neutral, and low income groups may even realise a net financial income via the scheme. However, for effective social distancing, trading must take place on thousands of spatio-temporally differentiated capacity slots, which is practically inconceivable.

Due to these limitations of individual methods, it is more likely that multiple demand management measures will have to implemented *simultaneously*.

A detailed quantitative comparison of the policies is out of the scope of this review article. However, the qualitative findings presented enable us to make the following policy recommendations. The use of inflow control and queueing systems seems inevitable to tackle extreme demand shocks and capacity fluctuations due to disruptions. An online, potentially smartphone based advance booking system has substantial potential in eliminating queueing. Advance booking could be arranged on an FCFS basis in off-peak periods, while peak slot demand can be regulated with monetary incentives. A slot reservation system also enables the operator to assign capacity to key workers and disadvantaged groups of travellers transparently. The revenue neutrality of peak spreading would improve its social support, and the literature offers surcharge-reward as well as tradeable permit schemes to achieve this.

## Data Availability

Not applicable.

## Demand management and economic efficiency

Marginal cost pricing rules are trivial when the social cost of travelling and its personal and external cost components are well defined in a public transport market. The forthcoming discussion reviews the impact of an explicit upper bound of ridership on optimal pricing rules.

Figure 2 combines two marginal personal benefit (MPB) curves with the marginal personal cost (MPC) and marginal social cost (MSC) of travelling, all expressed in function of *Q*, the volume of travellers in a static (time-invariant) scenario.^9^ The MPB functions quantify the personal benefit of individual passengers in a decreasing order; the economic literature often calls this the inverse demand or marginal willingness to pay function. The two demand curves can represent peak ($MPB_1$) and off-peak ($MPB_2$) scenarios, for example. The realised (equilibrium) demand depends on *p*, the price of travelling. It is assumed that only those passengers travel whose personal benefit is greater than the perceived price, including monetary and non-pecuniary user costs. Thus, $MPB_t(Q) = p_t$ is satisfied in equilibrium in each time period *t*. Without demand management interventions, the personal cost is the only element of the travel price, so that $MPB_t(Q) = MPC_t(Q)$, and equilibrium demand levels are $Q_1$ and $Q_2$ in the peak and off-peak markets.

The traditional argument supporting demand management interventions in welfare economics is that certain social costs of travelling are not perceived by passengers, while $MSC(Q) > MPC(Q)$. In this case, some of the realised trips generate less personal benefit than social cost, in the [$Q'_1$ , $Q_1$] interval, specifically. Theory suggests that if the price of travelling can be raised to $p'_1$, then capacity will be allocated to trips that satisfy the efficiency condition $MPB_1(Q) \ge MSC(Q)$. The efficiency-maximising peak demand level is $Q'_1 < Q_1$. Note, however, that the only objective of demand management in this case is to enhance efficiency, and its outcome might be undesirable according to other criteria such as social acceptance and political feasibility. In the off-peak, there is no major difference between the unmanaged and socially optimal demand levels, as $MPC \simeq MSC$.

With fixed capacity, the introduction of social distancing implies an exogenous upper bound on *Q*. Let us consider the upper bound indicated by the dashed vertical line in Figure 2. It is clear that social distancing might not require further demand management measures in certain low-demand markets. In the present case, $Q_2$ remains below the threshold. However, the social distancing constraint is binding in the peak. In this case, demand management measures are required to achieve two main policy objectives:

- *Quantitative goal:* Demand must be reduced from $Q_1$ or any other original equilibrium level to $Q''_1$, to comply with social distancing.
- *Allocative goal:* Depending on the exact demand management tool, the supplier may influence who can actually access the system among potential users, i.e. who is *selected* by the control intervention to travel.

The *quantitative goal* is a straightforward consequence of social distancing with fixed capacity. To make the *allocational* decision, a clear economic or alternative objective is required. Figure 2 follows the microeconomics tradition by sorting potential users according to their (decreasing) personal benefit from travelling. In this representation, if the allocational objective is to maximise efficiency, then only those potential users should travel, whose personal benefit is greater than $MPB(Q''_1)$. Pricing is one the most frequently proposed demand management techniques to implement this allocation. If the personal cost of travelling is increased to $p''_1$ by an appropriately set public transport fare, the demand restriction is implemented at maximum efficiency. Compared to the unpriced scenario when demand is $Q_1$, those passengers whose personal benefit lays between $MPB_1(Q_1)$ and $MPB_1(Q''_1)$ will be priced off the public transport service.

## Footnotes

1 Vickerman (2021) predicts major changes in the industrial organisation of public service provision, suggesting that “the deregulated, competitive model of transport may have to be confined to history” in countries that were previously in the forefront of liberalisation.

2 The Wells-Riley model is used to estimate the probability of infection in enclosed spaces. The infection probability is defined as a function of the number of infected persons, the quantum generation rate for infection as produced by one infected person, the pulmonary ventilation rate of susceptible persons, exposure time, and the ventilation rate of the space (Wells 1955).

3 As one of the anonymous referees has pointed out correctly, the subjective user valuation of personal health risks might not coincide with the objective risk, as the former can be influenced by the communication of public authorities, the media and other behavioural factors. It is clears that models of demand prediction must be based on subjective valuations to ensure precision. In the transport economics literature, externalities imposed upon fellow users are normally derived from subjective valuations, which is a debatable practice in case of incident probabilities that can be measured objectively as well. The present pandemic scenario may open up normative debates on this question.

4 Delgado et al. (2012) recall that the informal practice of a bus driver asking passengers not to board their heavily loaded vehicle but wait for next one is actually an intuitively motivated inflow control strategy. Such informal policies have been applied since the very beginning of public transport history.

5 High-quality information provision and effective media communication is indeed the cornerstone of the success of any demand management interventions in mass public transport.

6 Urgent trips of supposedly very high importance are actually made last-minute, so a negative correlation between early booking time and the net welfare effect of travelling cannot be excluded.

7 A smartphone based reservation system is already put in place in March 2020 at two busy stations of Beijing Subway, in response to the first wave of the Covid-19 outbreak. The primary goal of this scheme is to reduce station crowding. “After successfully making the reservations, passengers have a 30-minute window – from 10 minutes before the slot to 10 minutes after – to enter the stations via a fast track using a QR code generated on their phones. Those who do not make a reservation must wait in line before entering.” Source: ‘Beijing subway to pilot reservation system to control passenger flow’, China Daily, accessed on 29 September 2020

8 The history of tradeable credit programmes is rooted in environmental economics and pollution control, dating back to the seminal contributions of Dales (1968) and Montgomery (1972). Pollution oriented transport applications have remained present in the literature, see e.g. Wadud et al. (2008); Wadud (2011) and Han et al. (2017).
